# DNA Repair Gene Polymorphisms and Susceptibility to Urothelial Carcinoma in a Southeastern European Population

**DOI:** 10.3390/curroncol28030174

**Published:** 2021-05-14

**Authors:** Maria Samara, Maria Papathanassiou, Lampros Mitrakas, George Koukoulis, Panagiotis J. Vlachostergios, Vassilios Tzortzis

**Affiliations:** 1 Department of Pathology, Faculty of Medicine, School of Health Sciences, University of Thessaly, 41100 Larissa, Greece; msamar@uth.gr (M.S.); mpapat@uth.gr (M.P.); kougeo@med.uth.gr (G.K.); 2 Department of Urology, Faculty of Medicine, School of Health Sciences, University of Thessaly, University Hospital of Larissa, 41100 Larissa, Greece; lmitrak@uth.gr; 3 Department of Medicine, Division of Hematology and Medical Oncology, Weill Cornell Medicine, New York, NY 10065, USA

**Keywords:** DNA repair, XPC, ERCC2, ERCC2, XRCC3, single nucleotide polymorphism, urothelial carcinoma, bladder tumor

## Abstract

Single nucleotide polymorphisms (SNPs) in DNA repair genes may predispose to urothelial carcinoma of the bladder (UCB). This study focused on three specific SNPs in a population with high exposure to environmental carcinogens including tobacco and alcohol. A case-control study design was used to assess for presence of XPC PAT +/−, XRCC3 Thr241Met, and ERCC2 Lys751Gln DNA repair gene SNPs in peripheral blood from patients with UCB and healthy individuals. One hundred patients and equal number of healthy subjects were enrolled. The XPC PAT +/+ genotype was associated with a 2-fold increased risk of UCB (OR = 2.16; 95%CI: 1.14–4; *p* = 0.01). The −/+ and +/+ XPC PAT genotypes were more frequently present in patients with multiple versus single tumors (*p* = 0.01). No association was detected between ERCC2 Lys751Gln genotypes/alleles, and risk for developing UCB. Presence of the XRCC3 TT genotype (OR = 0.14; 95%CI:0.07–0.25; *p* < 0.01) and of the T allele overall (OR = 0.26; 95%CI:0.16–0.41; *p* < 0.01) conferred a protective effect against developing UCB. The XPC PAT −/+ and XRCC3 Thr241Met SNPs are associated with predisposition to UCB. The XPC PAT −/+ SNP is also an indicator of bladder tumor multiplicity, which might require a more individualized surveillance and treatment.

## 1. Introduction

Urothelial carcinoma of the bladder (UCB) is the tenth most common cancer worldwide with an increasing incidence [1]. Τhe highest rates of UCB worldwide are found in North America and in countries of Western and Southern Europe [1,2]. Greece shows the highest age-standardized incidence rate of UCB per 100,000 men (26.5) [2].

The use of next generation sequencing (NGS) technology has deepened out understanding of the molecular landscape of UCB tumors [3]. Through an integrated analysis of DNA mutations, RNA expression profiles and subtype classification, and epithelial-mesenchymal and immune infiltrate signatures, common molecular alterations in genes such as FGFR3, CD274 (PD-L1), and others were not only identified as key phenotypic elements of UCB but were also placed within a framework of potential responses to different therapies [3].

Further to this improved understanding of the somatic contexture of UCB tumors, heritable common gene variants of low-penetrance, with a risk allele frequency more than 5% and odds ratio less than 1.5 [4], also known as single nucleotide polymorphisms (SNPs), have been increasingly recognized as potential contributors to urothelial carcinogenesis [5]. A well-described mechanism involves the detrimental effect of environmental risk factors like smoking on DNA repair, resulting in accumulation of oncogenic mutations [6]. Tobacco contains more than 60 carcinogens including benzidine derivatives and aromatic amines. These substances have an important role in developing UCB [7,8]. Smokers have a 4–7-fold increased risk of developing UCB compared to non-smokers, and cigarette smoke is considered the most important risk factor for UCB development [9]. Moreover, cigarette smoking increases the risk of recurrence and progression of non-muscle-invasive bladder cancer (NMIBC) [10]. Alcohol consumption may also increase the risk of UCB, particularly in males [11]. The presence of SNPs in key DNA repair pathways that are involved in the response to DNA damage caused by these environmental carcinogens, including the nucleotide excision repair (NER) and the homologous recombination repair (HRR) pathways, may increase the risk of developing UCB [6,12]. For some genes within these pathways, e.g., ERCC2 (NER), the correlation of specific SNPs with cancer risk has been inconsistent across different studies [13]. Other SNPs in genes such as XPC (NER) and XRCC3 (HRR) have a more pronounced effect in specific populations or require validation in large single studies due to biases in meta-analyses of smaller studies [14,15].

In this study, we examined the incidence of three common NER and HRR gene SNPs, namely, XPC PAT +/−, ERCC2 Lys751Gln, and XRCC3 Thr241Met, in a Greek population with a high environmental exposure to tobacco and alcohol. We assessed potential associations of these SNPs with the risk of developing UCB and its histopathological features.

## 2. Materials and Methods

### 2.1. Study Population

Patients with a histological diagnosis of UCB were prospectively enrolled in the study. Patients harboring tumors with histological variants or upper tract location were excluded. Healthy men and women > 18 years of age were also enrolled and served as control group. The study was approved by our Institutional Review Board and Ethics Committee and is in accordance with the declaration of Helsinki, as revised in 2013. An informed consent was obtained from each subject before study entry. Clinical and pathological characteristics of patients were recorded, including age, sex, family history of UCB or other malignancy, smoking status (active, former, or never smoker), and alcohol use (non-drinker, light to moderate drinker, heavy drinker). Peripheral blood from healthy subjects and patients was tested for the presence of XPC PAT +/−, XRCC3 Thr241Met, and ERCC2 Lys751Gln SNPs.

### 2.2. Genomic DNA Extraction

Enrichment of tumor cells up to 70% was performed after microdissection on hematoxylin and eosin-stained sections of tumor specimens reviewed by a pathologist. 5–10 sections of 10 μm thickness from FFPE samples were used and DNA extraction was performed according to the manufacturer’s protocol (Invitrogen Genomic DNA Mini Kit, Thermo Fisher Scientific, Loughborough, UK). Genomic DNA from peripheral blood samples was also extracted with Purelink Genomic DNA (Invitrogen Genomic DNA Mini Kit, Thermo Fisher Scientific), according to the manufacturer’s instructions. DNA was eluted in distilled water and quantified by both agarose gel electrophoresis and absorption spectrometry at 260/280 nm.

### 2.3. SNP Genotyping

Single nucleotide polymorphisms (SNPs) in DNA repair genes ERCC2 (rs13181) and XRCC3 (rs861539) were determined by the polymerase chain reaction-restriction fragment length polymorphism (PCR-RFLP) method. Genomic DNA was also amplified for intron 9 of the XPC gene (PAT +/− insertion). The amplification mixture consisted of 5 μL of 10X reaction buffer, 2.5 mM MgCl2, 1.6 mM dNTPs, a 0.1 μM concentration of each primer, 1u Taq DNA Polymerase (ThermoFisher Scientific Inc., Loughborough, UK) and 5 μL of template DNA in a final volume of 50 μL. Amplification conditions are described in Table S1. A non-template control (NTC) was included in all PCR reactions. Primer sequences for each SNP or PAT insertion are presented in Table S2.

### 2.4. Restriction Fraction Length Polymorphism (RFLP) Assay

PCR products for ERCC2 rs13181 and XRCC3 rs861539 were subjected to RFLP analysis using the restriction enzymes PstI and NcoI (10,000 U), respectively (New England Biolabs Inc, Hitchin, UK). Then, 10 μL of each PCR product was added in 3 μL of digestion buffer along with 15 units of PstI or NcoI, respectively. Distilled water was added to a final volume of 30 μL. Digestion products for rs13181 and rs861539 are shown in Table S3.

### 2.5. Statistical Analyses

Statistical analysis was performed with IBM SPSS v22 software. Pearson’s chi square was used to evaluate the association of each polymorphism with UCB risk, using the recessive model for the XPC PAT +/− and ERCC2 Lys751Gln SNPs [(+/+) versus (−/−) and (−/+), (Gln/Gln) versus (Lys/Lys) and (Lys/Gln)]. The dominant model was used for XRCC3 Thr241Met [(Thr/Met) and (Met/Met) versus (Thr/Thr)] with 95% confidence intervals. The strength of association between SNPs in tumor tissues was measured by odds ratios (OR) and relative risks (RR) with 95% confidence intervals (CI). The *p* values < 0.05 were considered significant.

## 3. Results

We examined 100 patients with UCB and 100 healthy subjects (control group). The majority of patients (89%) were males, and 11% were females. The median age of the group was 70 years (range: 45–92) (Table 1). The control group consisted of 50 males and 50 females, at a median age of 30 years (range 25–35), without any prior history of cancer.

Approximately one third of patients were active smokers (≥20 pack-years) and 49% were former smokers who had quit within 5 years from study entry (Table 1). With respect to alcohol consumption, patients were categorized as non-drinkers (29, 29%) and drinkers (71, 71%). Drinkers were further characterized as light-to-moderate drinkers, consuming <3 three servings of alcohol per week (29, 43%), and heavy drinkers, consuming > 5 servings of alcohol per week (38, 57%) (Table 1).

Fourteen patients (14%) had a family history of at least one first-degree relative with any type of cancer (Table 1). The majority of patients had non-muscle invasive disease, including 45% Ta and 35% T1 tumors. Fifty-nine patients had a single tumor, whereas 41 had multiple tumors. At the time of study, 71 individuals were diagnosed with primary UCB while 19 had recurrent disease (Table 1).

We found that the intronic XPC PAT −/− genotype was less frequent in the patients’ group compared to the control group whereas the −/+ and +/+ genotypes were enriched in patients compared to healthy subjects (*p* = 0.012, OR = 1.15, 95% CI = 0.78–1.69) (Table 2). The +/+ genotype was associated with a 2-fold increased risk of UBC (OR = 2.16; 95% CI: 1.14–4; *p* = 0.01) (Table 2). Conversely, the XRCC3 CC genotype was more frequent in patients versus healthy controls, while the opposite was the case for CT, TT genotypes (Table 2). Presence of the XRCC3 TT genotype (OR = 0.14; 95%CI:0.07–0.25; *p* < 0.001) and of the T allele overall (OR = 0.26; 95%CI:0.16–0.41; *p* < 0.001) was associated with a significantly lower UBC risk (Table 2). There was no association between the ERCC2 genotypes and risk for developing UBC.

We then sought to address whether the XPC PAT −/+ and the XRCC3Thr241Met SNPs follow any particular phenotypical pattern with respect to patients’ histopathological characteristics, including grade, stage, recurrent disease, and number and diameter of bladder tumors. The XPC PAT SNP was associated with the number of tumors. In particular, the −/− genotype was associated with the presence of a single tumor rather than multiple bladder tumors in patients (OR = 15.38, 95% CI = 1.953–121.163; *p* = 0.01) while the −/+ and +/+ genotypes were more frequently present in patients with multiple tumors (Table 3). No other significant correlations were observed between XPC PAT −/+ or XRCC3 Thr241Met SNPs and the rest histopathological characteristics.

## 4. Discussion

This case-control study examined the incidence of XPC PAT +/−, XRCC3 Thr241Met and ERCC2 (ERCC2) Lys751Gln DNA repair gene SNPs as well as their possible associations with the risk of developing UCB within a Greek population. The XPC PAT +/+ genotype was associated with a 2-fold increased risk of UCB, whereas the XRCC3 TT genotype and T allele exerted protective effects.

Our findings are in line with prior candidate gene studies and meta-analyses in other (non-Greek) populations, supporting a role of these SNPs in predisposing to UCB. The PAT +/+ genotype was previously shown to confer a significantly higher risk of developing UCB compared to the PAT −/− genotype [14], as opposed to the protective effect of the XRCC3 TT genotype [16]. Taking a step further, we also provide evidence that presence of the +/− or +/+ XPC PAT genotypes may be linked to a more aggressive phenotype consisting of multiple bladder tumors, compared to the −/− genotype which was more common in single tumors. If confirmed in larger studies, this finding may have therapeutic implications, suggesting the need for an earlier and perhaps more intensive intravesical therapy in such cases. In a broader context, it is plausible to hypothesize that patients with SNPs compromising DNA repair function could result in higher sensitivity to systemic therapies including platinum chemotherapy or/and immune checkpoint inhibitors, extrapolating from studies conducted in patients with somatic DNA repair defects [17,18]. Particularly for XPC, lessons learned from other tumors such as ovarian carcinoma suggest that the XPC poly (AT) (PAT) (−/+)/ (−/−) genotype versus the (+/+) genotype is associated with a prolonged progression-free survival after treatment with platinum-based chemotherapy (17 months versus 11.6 months) [19]. Likewise, the presence of ERCC2 rs13181 and XRCC3 rs861539 SNPs is significantly associated with treatment-related outcomes, including progression-free and overall survival for ERCC2 and objective response rate for XRCC3, respectively, in non-small cell lung cancer patients receiving platinum-based regimens [20].

We did not identify any significant correlation of the ERCC2 Lys751Gln SNP with UCB risk in our studied population. Although other SNPs, such as Asp312Asn (rs1799793 G > A), were consistently reported to correlate with increased predisposition to UCB [21,22,23,24,25], studies on Lys751Gln have yielded discordant results. Zhu et al. [21] reported that Lys751Gln is associated with UCB risk in their overall population and in Caucasians, using a recessive model. Similarly, Li et al. [22] demonstrated a positive association between Lys751Gln and UCB risk regardless of Asian or Caucasian origin of patients. Other studies in Asian and Caucasian populations have argued against a significant role of this SNP in developing UCB [23,24,25,26]. Inherent differences among negative and positive studies in the design, population descent, and size may, at least partially, account for ambiguous findings on the role of ERCC2 Lys751Gln SNP.

The strengths of our prospective study include focus on a specific ethnic population, not studied before, with a high environmental risk of UCB pertinent to tobacco and alcohol exposure, and the investigation of correlations with disease characteristics further to UCB risk. The main limitations were the rather small sample size, lack of age-matched controls, occupational history, and composite risk score calculation including environmental risk contributions (tobacco, alcohol).

## 5. Conclusions

In conclusion, the XPC PAT +/+ and XRCC3 TT SNPs may predispose to and protect from UCB, respectively, in a Greek population with significant environmental exposure to tobacco and alcohol. The XPC PAT +/+ and −/+ SNPs are also indicators of bladder tumor multiplicity, which might require a more individualized surveillance and therapeutic approach in such patients. Collectively, our findings may help predict susceptibility to bladder carcinogenesis and adopt personalized screening strategies in these SNP carriers.

## Figures and Tables

**Table 1:** Clinical and pathological characteristics of UCB patients.

Characteristic		Patients	
		*n*	%
Gender	Males	89	89
	Females	11	11
Age (years)	Mean ± SD	69.9 ± 10.7	
	Range	45–92	
Grade	G0	2	2
	G1	22	22
	G2	31	31
	G3	45	45
Stage	Ta	45	45
	T1	35	35
	T2	20	20
Tumor Multiplicity	Single	59	59
	Multiple	41	41
Tumor size (cm)	≤2	42	42
	>2	58	58
Disease Status	Primary	71	71
	Relapse	19	19
Family history	Yes	14	14
	No	86	86
Smoking	Never	21	21
	Former	49	49
	Active smokers	30	30
Alcohol use	Non-drinkers	29	29
	Drinkers	71	71
	Moderate drinkers	30	42
	Heavy drinkers	41	58

**Table 2:** Frequencies of studied DNA repair gene SNPs in patient and control groups.

SNPs	Patients	%	Controls	%	χ^2^ Test	*p* Value	OR (95% CI)	*p* Value
ERCC2 rs13181								
TT	24	24	39	36				
TG	58	58	52	49				
GG	18	18	16	15	3.4	0.179		
G allele	94	47	84	39				
T allele	106	53	130	61	3.4	0.064	0.72 (0.49–1.08)	0.11
XPC PAT −/+								
−/−	19	19	36	34				
−/+	55	55	35	32				
+/+	26	26	36	34	11.3	0.04	2.16 (1.14–4)	0.01
-	93	46.5	107	50				
+	107	53.5	107	50	6.2	0.13	1.15 (0.78–1.69)	0.47
XRCC3 rs861539								
CC	79	79	36	34				
CT	14	14	59	55				
TT	7	7	12	11	45.9	<0.01	0.14 (0.07–0.25)	<0.001
C allele	172	86	131	65.5				
T allele	28	14	83	34.5	43.9	<0.01	0.26 (0.16–0.41)	<0.001

**Table 3:** Association of XPC PAT −/+ SNP with the number of bladder tumors per patient.

XPC PAT Genotype	Single Tumor	Multiple Tumors	*p* Value
−/−	17	1	
−/+ and +/+	42	38	0.01

## Data Availability

The data presented in this study are available within the article and Supplementary Material.

## Supplementary Materials

The following are available online at https://www.mdpi.com/article/10.3390/curroncol28030174/s1, Table S1: Polymerase chain reaction (PCR) amplification conditions, Table S2: 5′→3′ primer sequences for each SNP studied, Table S3: Genes, SNPs, restriction enzymes used, and sizes of genotypes in base pairs (bp).
