# Supplementary material for: DNA Repair Gene Polymorphisms and Susceptibility to Urothelial Carcinoma in a Southeastern European Population
Source: Curr Oncol. 2021 May 14;28(3):1879–85. doi: 10.3390/curroncol28030174 (PMC8161783; doi:10.3390/curroncol28030174)
Supplement: Supplementary file 1 [file curroncol-28-00174-s001.zip › curroncol-1201603-supplementary.pdf]

Supplementary Table 1. Polymerase chain reaction (PCR) amplification conditions.

PCR program	Temperature (ºC)	Time (minutes)	Cycles
Denaturation	94	05:00	1
Amplification	94	00:30	35
	64	01:00	
	72	01:00	
Extension	72	10:00	1
Cooling	4	∞	

Supplementary Table 2. 5’→ 3’ primer sequences for each SNP studied.

SNP	Primer	5’ – 3’ sequence
XPC PAT +/-	Forward	TAGCCAGCAGTCAAAG
	Reverse	TGTGAATGTGCTTAATGCTG
XRCC3 Thr241Met	Forward	GCCTGGTGGTCATCGACTG
	Reverse	CAGGGCTCTGGAAGGCACTGCTCAGCTCACGCACC
XPD Lys751Gln	Forward	GCCCGCTCTGGATTATACG
	Reverse	CTATCATCTCCTGGCCCCC

Supplementary Table 3. Genes, SNPs, restriction enzymes used, and sizes of genotypes in base pairs (bp).

Gene	SNP	RefSeq ID	Restriction Enzyme	Wild type genotype	SNP genotype
XPD	rs13181	NM_000400.3: c.2251A>T	PstI	290 + 146 bp	229+ 146+ 63 bp
XRCC3	rs861539	NM_005432.3: c.722C>T	NcoI	136 bp	97+ 39 bp
